# The role of patient and public involvement and engagement (PPIE) within the development of the EQ Health and Wellbeing (EQ-HWB)

**DOI:** 10.1186/s41687-022-00437-y

**Published:** 2022-04-08

**Authors:** Jill Carlton, Tessa Peasgood, Clara Mukuria, Julie Johnson, Margaret Ogden, Wade Tovey

**Affiliations:** 1 School of Health and Related Research (ScHARR), University of Sheffield, Regent Court, 30 Regent Street, Sheffield, S1 4DA UK; 2 Melbourne School of Population and Global Health, University of Melbourne, Melbourne, Australia; 3 On Behalf of the EQ-HWB PPIE Group, Sheffield, UK

## Abstract

**Objectives:**

The value of patient and public involvement and engagement (PPIE) within the development and refinement of outcome measures is becoming increasingly recognized. The aim of this paper is to provide an overview of how PPIE was integrated within the development of a new measure designed for use in economic evaluations across health and social care, the EQ Health and Wellbeing (EQ-HWB™).

**Methods:**

Four PPIE sessions were held at key stages. Discussions from each session and the outcome of any tasks were shared with the wider research team and used to help inform decision-making.

**Results and discussion:**

PPIE covered several components of outcome measure development including; review of conceptual model; discussion on sub-domain inclusion; item refinement and reduction; pre-testing of items; selection of items for the measure; and design of the measure. Key learning points for future projects were highlighted including; consideration of practicalities, resources and logistics of PPIE activities; how sessions and activities are managed effectively; and how to managing expectations and communication from both researcher and PPIE perspectives.

**Conclusions:**

The PPIE group provided invaluable insight into perspectives of future patients and carers. Their input was fed into a number of developmental stages. The formal involvement from the PPIE group meant that the voice of the general public was heard. This helped ensure the appropriateness of the design of the final measure.

## Background

The value of patient and public involvement and engagement (PPIE) within the development and refinement of outcome measures and preference-based measures is becoming increasingly recognized [1–5]. Guidance exists to help researchers plan and conduct meaningful PPIE [6–9]. There remains a paucity of documentation for this important component, and shared learning from such activities is often overlooked. The aim of this report is to provide an overview of how PPIE was integrated within the development of a new measure, the EQ Health and Wellbeing (EQ-HWB™) using an existing framework [4]; to describe the activities used to facilitate discussion and decision-making; and to reflect upon our experiences, using the GRIPP2-SF checklist [10].

### The project

**Fig. 1:**
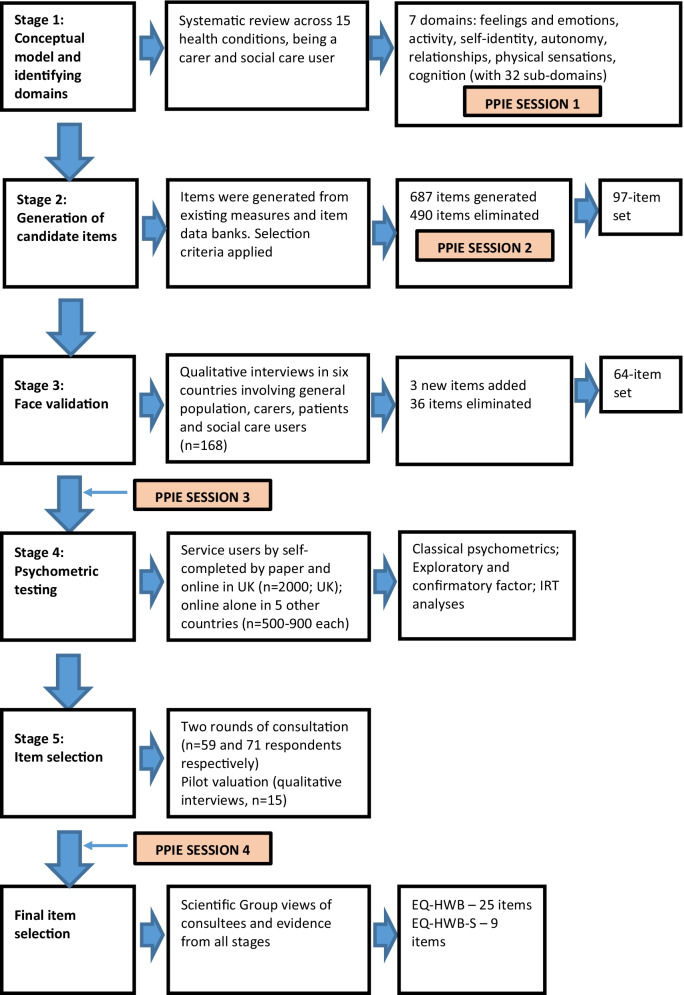
E-QALY project stages

The international Extending the QALY (E-QALY) project was initiated to develop a measure which captures aspects of quality of life or wellbeing for use in economic evaluations across health and social care. The project encompassed several stages (Fig. 1). Further details of the project are described elsewhere [11].

### The PPIE group

PPIE members were recruited via several sources. We sought members of the public with a health condition (not specified), or informal carers of adults over 18 years. Firstly, an advertisement on the National Institute for Health Research (NIHR) INVOLVE website designed to advertise opportunities for public involvement in the National Health Service (NHS), public health and social care research (https://www.peopleinresearch.org/) recruited four PPIE members. Secondly, the lead PPIE Officer of the School of Health and Related Research (ScHARR), University of Sheffield approached individuals with previous PPIE experience (n = 2). Finally, one of the stakeholder groups of the EQALY project (National Institute for Clinical Excellence, NICE) approached existing PPIE representatives from within their own network (n = 1). The PPIE group membership consisted of seven members, who had experienced a range of health conditions, both personally and/or in a caring capacity (for a family member).

### Activities

Four face-to-face PPIE sessions were held at key stages (Table 1).

**Table 1:** Detail of each PPIE session

	Session 1	Session 2	Session 3	Session 4
**EQALY stage**				
Aim	Review of conceptual model to ensure validity	Discussion on sub-domain inclusion Item refinement and reduction	Item reduction Pre-testing of items	Selection of items for the measure Design of the measure
Participants	7	7	5	6
**Methods**				
Preparation material sent in advance	✓	✓	✓	✓
Background introduction and/or recap	✓	✓	✓	✓
***Tasks***				
Individual assessment			✓	
Paired assessment	✓			✓
Group discussion	✓	✓	✓	✓
Sorting task		✓	✓	✓
Outcomes	Agreement with proposed themes and subthemes	Suggestion of dropping items to take forward into next stage of the project	Advice on which items to keep/drop, wording of items	Advice on final selection of items, wording of items, order of items within the measure, instructions for completion, and overall layout
Follow-up	✓	✓	✓	Shared in the meeting
PPIE quotes	“The experience of PPI in the EQALY project was valuable to me. This was the first time I’d been involved in measure development. I was able to give my views on what constitutes well-being and quality of life for a patient living with comorbidities. That this study spanned health, public health & especially social care, was an added bonus—I had recently experienced social care as both patient & carer” “It was the range of PPI activities and equality of opportunity which impressed me greatly. There were four face to face meetings which were very interactive. Important outputs were envisaged. Achieving those outputs as the study progressed, turned out to be dynamic & fun” “I think the opportunity for reflection was a strong theme of what was offered to the PPIE members—there was a sense of it not being hurried—but it progressed in a very considered way”			

One member of the group withdrew from the project after session 2 due a change in personal circumstances. One member was unable to attend session 3 due to ill health

#### Session 1

The aim of Session 1 was to obtain feedback and input on the results of Stage 1. As this was the first time the PPIE group had convened it was felt important to include a ‘getting to know each other’ exercise to make members feel more comfortable and to encourage discussion and interactions. This was followed by a brief introduction to the overall aim of the project, including an explanation of the structure of the teams and governance groups; the role of the PPIE group, expectations; and what their involvement would be over the course of the project.

The main activity of the session was to reflect upon the findings of Stage 1 [12]. High-level themes and potential subthemes for the new measure identified through the review were shown (and described) to the group. Members were asked to work in pairs to consider each of the themes/subthemes including whether they should be included or merged with other subthemes. Members were encouraged to include any comments to explain their decision or thought process. Each theme was considered for approximately 15 min, followed by feedback in a wider group discussion. Results were collated and distributed to PPIE members after the meeting for member checking [13, 14]. Members were encouraged to add any additional thoughts or comments having had further time to reflect on the task. Results were used to inform the final selection of themes and subthemes [12].

#### Session 2

Members were updated on the project including which of the potential themes/subthemes raised in the Session 1 had been taken forward and why. The process for identifying potential items and criteria for judging the best item were introduced. The group then discussed the draft items from Stage 2 arranged into themes/sub-themes and considered these from the perspective of a respondent in a future study [15]. They discussed issues ranging from ambiguous interpretation, translation, intrusiveness, social desirability and ease of completion. This discussion supported refinement and in some cases dropping of items. The research team’s choice to present items without the response choices (which had yet to be agreed) nor in a questionnaire format resulted in considerable confusion, which hindered the efficiency of this meeting. Results informed the item selection process for Stage 2.

#### Session 3

Session 3 began with a presentation of the key findings of the face validity studies (Stage 3) conducted in the UK and internationally [15]. Some examples were provided to show which items had been excluded for consideration, with reasons behind their exclusion. The aim of Session 3 was to obtain members’ views on potential item selection. This was facilitated by a practical exercise where each domain was considered in turn. Potential items were presented on coloured cards, with different colours used for each domain. The group were asked to discuss and allocate items to one of three categories; *include*, *reject* or *undecided*. Participants moved around the room, and placing the items on boards for each of the aforementioned categories. After each domain, members had the opportunity to reflect upon their decisions, and were given an opportunity to change the allocation of items. The final allocation of items were noted alongside discussions that were observed. The use of colours to reflect the different domains helped members to think about whether the proposed items adequately covered the overarching themes and the conceptual model for the measure. Results (Table 2) were used alongside other evidence in consultation regarding which items to include for the EQ-HWB [11].

**Table 2:** Results of PPIE views of draft items for consideration for the EQ-HWB measure

Item	Reject	Undecided	Include
**Domain: activity**			
I enjoyed what I did (F)	✓		
I was able to do the things I value (F)	✓		
I could do the things I wanted to do (F)			✓
I was able to do what I needed (F)	✓		
How well were you able to do your day to day activities (e.g. working, shopping, travelling) (D)			✓
My personal needs were met (e.g. being washed, going to the toilet, getting dressed, having food when I needed) (F)		✓	
Given the help I had/received my self-care needs were met (e.g. being washed, going to the toilet, getting dressed, having food when I needed)		✓	
I was able to look after myself (F)	✓		
I was able to look after myself (e.g. being washed, going to the toilet, getting dressed, having food when I needed) (F)			✓
I was able to get around inside my home with no difficulty (D)			✓
I was able to get around outside with no difficulty (D)			✓
Because of hearing and/or speech, how difficult did you find it to have a conversation (D)		✓	
How well can you hear (using hearing aids if you usually wear them) (D)			✓
How well can you see (using your glasses or contact lenses if they are needed) (D)		✓	
I was able to do the things I wanted to do (S)			✓
**Domain: autonomy**			
I felt able to cope with my day to day life (F)			✓
I felt unable to cope with my day to day life (F)	✓		
I felt overwhelmed by the problems or situation (F)			✓
I felt in control of my daily life			✓
I felt I had no control over my day to day life (F)	✓		
**Domain: cognition**			
I found it hard to concentrate (F)			✓
I found it hard to pay attention (F)	✓		
I had trouble thinking clearly (F)	✓		
I had trouble remembering (F)	✓		
I felt confused (F)	✓		
**Domain: feelings and emotions**			
I felt happy (F)			✓
I felt unhappy (F)	✓		
I felt sad (F)			✓
I thought my life was not worth living (F)	✓		
I felt that I had nothing to look forward to (F)	✓		
I felt frightened (F)		✓	
I felt afraid (F)	✓		
I felt safe (F)			✓
I felt unsafe (F)	✓		
I felt anxious (F)			✓
I felt worried (F)	✓		
I felt calm (F)		✓	
I felt irritable (F)			✓
I felt angry (F)		✓	
I felt frustrated (F)			✓
I lost my temper easily (F)		✓	
I felt cheerful (F)	✓		
**Domain: physical sensations**			
I had no physical pain (mild pain etc.) (S)			✓
How often do you experience physical pain (F)			✓
I had no physical discomfort (mild discomfort etc.) (S)			✓
How often do you experience physical discomfort (F)			✓
I felt exhausted (F)			✓
I felt very tired (F)			✓
I had problems with my sleep (F)			✓
**Domain: Relationships**			
I felt unsupported by people (F)	✓		
I had support when I needed it (F)			✓
I got along well with people around me (F)			✓
I felt lonely (F)			✓
I felt there was nobody I was close to (F)	✓		
I felt I had no one to talk to (F)	✓		
I felt isolated (F)		✓	
I felt people avoided me (F)		✓	
I felt accepted by others (F)		✓	
I felt excluded (F)			✓
I felt left out (F)		✓	
**Domain: self-identity**			
I felt confident in myself (F)			✓
I felt unsure about myself (F)	✓		
I felt good about myself (F)	✓		
I felt like a failure (F)			✓

*F *frequency response option, *S *severity response options

#### Session 4

The aim of the session was to inform the final layout and presentation of the measure. Much consideration was given to the instructions on how respondents complete it. Members were asked for views on the ordering of items within the questionnaire. Laminated cards were used to help members visualize what the questionnaire could look like. This was then produced in a mock format, after which further feedback was given on the layout. Members provided views on whether items should be alternately shaded (for reading ease), whether items should be numbered, and spacing between the items. They expressed that the layout should incorporate colour, and that the final format of the questionnaire should be of high-quality production, such that it would suggest it was an important thing for respondents to complete. Members had mixed views about the exact order of items although agreed that the opening item should be easy to complete, and final items should minimise the risk of leaving respondents feeling negative emotions. Members were keen to ensure that the title of the measure, and any invite to complete the measure, should clearly communicate what is being collected and why. The draft version was modified with members’ input then printed and shared at the meeting. In addition, they advocated for clear instructions on which perspective the respondent completing the measure should approach it from, i.e. carers complete it in relation to themselves, and not on behalf of the person they care for. Members also contributed the perspective of future respondents completing in English, where this is not their first language, and highlighted potential ambiguities and difficulties they may face with interpretation. This is a good example of the PPIE group reminding researchers to communicate in plain language. The feedback contributed to the layout of the experimental version of the EQ-HWB.

A list of activities and key inputs from the PPIE group are shown in Table 1. The write-up from each session was shared with the broader project team and formed part of the body of evidence that was used in decision making at each stage of the project. A summary of engagement across each of the development stages is shown in Table 3.

**Table 3:** PPIE engagement for each stage of development within Extending the QALY project

Stages of measure development [4]	PPIE included?
1. Establishing a need for a new or refined measure	✗
2. Development of a conceptual model	✓
3. Identifying item content	✓
4. Item development	✗
5. Item reduction (and refinement)	✓
6. Pretesting of items (cognitive interviews/debriefing)	✓
7. Psychometric survey design	✗
8. Psychometric survey analysis	✗
9. Selection of items for the measure	✓
10. Design of the measure	✓
11. Dissemination of the measure	✓

## Discussion

The role of PPIE in the Extending the QALY project was very valuable, both in terms of helping to develop the EQ-HWB, but also in providing experience for the research team in how to best integrate and conduct PPIE more broadly [16–18]. There are several aspects worth highlighting that we would advocate others to consider when planning PPIE for developing measures.

### Practicalities, resources and logistics

Existing literature and guidance documents clearly state the importance of good planning for PPIE activities [4, 18, 19]. We found this to be challenging, particularly when considering the timing of PPIE meetings within the project stages when important outputs of the meeting were required. Practical considerations such as ensuring meeting rooms were fully accessible to people of all physical abilities, ease of access to other facilities (such as prayer rooms), location of the buildings and links to public transport were all important activities requiring time [19, 20]. It was also necessary to allocate enough resource to the design, preparation and circulation of both pre-reads and session materials [4, 21].

### Managing the sessions and activities

We found it beneficial to have several members of the research team attend PPIE meetings. This supported smooth running of the sessions as well as administrative logistics. Delays in reimbursing out of pocket expenses can compromise working relationships [22]. Having several members of the research team present allowed for us to engage with the PPIE members during the breaks. This helped foster good relationships within the group, and facilitated an environment where members felt valued and comfortable to express their views openly [20, 23]. Having a number of researchers present allowed for a change in personnel to lead aspects both within and between sessions. This proved beneficial when explaining terminology and/or the tasks.

We noted the sessions became more productive and fruitful as the project progressed. During the early sessions the concepts and visualization of the “end product” was challenging for the PPIE members. There were lots of discussion of issues that, whilst very important (often relating to implementation of measures within the real-world setting), were outside of the remit of the E-QALY project. Frustrations were felt from both PPIE members and researchers, and there was a sense of ‘lack of purpose’, particularly during Sessions 1 and 2. We noted the PPIE members became very absorbed when presented with a “task” versus a more general discussion. This was particularly apparent during Sessions 3 and 4. Here members could start to see the questionnaire beginning to take shape and felt that they could see the merit of their involvement within the development process. We would strongly advocate researchers to think of ways to integrate and incorporate task-based exercises in order to facilitate discussions [23]. We found this to be very useful, particularly when demonstrating the impact of decision-making on the length (i.e. number of items) of the proposed measure.

### Managing expectations and communication

The sessions themselves are very demanding. From a researcher perspective, it would have been preferable for the meetings to last longer. The meetings felt very concentrated and intense with many issues to cover. Each meeting started with a recap and debrief as to what stage the overall project was at. Whilst it was tempting to rush this component, trying to do so proved counterproductive. It resulted in the group seeking additional clarification later in the session. It was very challenging to cover everything within the scheduled time that was available. It was important that sessions finished on time due to work, travel and/or caring commitments. Comfort breaks were included within each session [20]. Each one of the sessions ended with a feeling that more could have been achieved.

When aspects of the overall project became delayed, this resulted in periods where limited communication with the PPIE members occurred. We were fortunate that our inactions did not lead to member disengagement, however this is certainly a possibility when poor communication can result in members feeling undervalued or their input viewed as ‘tokenistic’. We endeavoured to inform PPIE members about the stages (and results) of the overall project, and when we anticipated that we would wish to reconvene with them. However, PPIE members noted a concern with lack of regular contact.

Another aspect of communication is that of feedback. Whilst we did ask for feedback following each session, this was relatively informal. It was useful to consider areas that worked well as well as to identify issues that could be improved for subsequent sessions. Location of the meetings (i.e. closer to public transport links) was one example that led to a positive change for subsequent sessions. Researchers need to learn about the most efficient way of incorporating PPIE when developing measures. The opportunity to seek more formal evaluation of the PPIE process was not maximized during the project, but should be encouraged.

One of the roles of PPIE is to formally incorporate lived experiences to research. In this project our PPIE group membership was small, and it possible that a larger group with wider representation across different health conditions may have resulted in different decisions, particularly in Session 3. It is plausible a person who has not experienced difficulties with specific items (or concepts) may not rank its importance as highly as more personally relevant items. Furthermore, despite the introduction and discussion of scope of the project being outlined in Session 1, it is possible that members brought their own interpretations of health and wellbeing that did not align with domains/themes provided in the EQ-HWB. Whilst this is not a limitation per se, it is feasible that individual’s definitions of health and wellbeing may have affected the relative importance of any items (Session 3). There is a balance to be struck between ensuring a suitable number of PPIE members required to ensure breadth of experiences, and practicalities of group sessions.

## Conclusion

Throughout the development of the EQ-HWB measure, the PPIE group provided invaluable insight into perspectives of future patients and carers. Their input fed into a number of developmental stages, helping to ensure the appropriateness of the design and content of the final measure.

## Data Availability

Not applicable.
